# Functional and diffusion tensor magnetic resonance imaging of the sheep brain

**DOI:** 10.1186/s12917-015-0581-8

**Published:** 2015-10-14

**Authors:** Wonhye Lee, Stephanie D. Lee, Michael Y. Park, Lori Foley, Erin Purcell-Estabrook, Hyungmin Kim, Seung-Schik Yoo

**Affiliations:** Department of Radiology, Brigham and Women’s Hospital, Harvard Medical School, Boston, MA USA; Invasive Cardiovascular Experimental Laboratory, Brigham and Women’s Hospital, Harvard Medical School, Boston, MA USA; Center for Bionics, Korea Institute of Science and Technology, Seoul, Korea

**Keywords:** Sheep, Sensorimotor, Visual, fMRI, DTI, Tractography

## Abstract

**Background:**

An ovine model can cast great insight in translational neuroscientific research due to its large brain volume and distinct regional neuroanatomical structures. The present study examined the applicability of brain functional magnetic resonance imaging (fMRI) and diffusion tensor imaging (DTI) to sheep using a clinical MR scanner (3 tesla) with a head coil. The blood-oxygenation-level-dependent (BOLD) fMRI was performed on anesthetized sheep during the block-based presentation of external tactile and visual stimuli using gradient echo-planar-imaging (EPI) sequence.

**Results:**

The individual as well as group-based data processing subsequently showed activation in the eloquent sensorimotor and visual areas. DTI was acquired using 26 differential magnetic gradient directions to derive directional fractional anisotropy (FA) and apparent diffusion coefficient (ADC) values from the brain. White matter tractography was also applied to reveal the macrostructure of the corticospinal tracts and optic radiations.

**Conclusions:**

Utilization of fMRI and DTI along with anatomical MRI in the sheep brain could shed light on a broader use of an ovine model in the field of translational neuroscientific research targeting the brain.

## Background

Ovine models for neurological application targeting the brain have been gaining momentum for providing translational information on therapeutic applications to humans. Compared to small animal testing [1], the ovine model of medical research provides important translational information that contributes meaningful insights to human studies. Unlike pigs, which have a flat and thick skull [2], sheep have a relatively round skull that provides comparable structural proximity to the human skull. With large brain volume and distinctive neuro-anatomical structures, the use of the ovine models has been continuing to expand in neuroscience research fields, for example, stroke [3], epilepsy [4], and brain injury models [5]. Emerging therapeutic applications, such as non-invasive focused ultrasound surgeries to ablate soft/tumor tissue [6, 7], would also benefit from using the ovine model.

Anatomical and functional neuroimaging techniques are widely used to characterize functional-structural correlates or any morphological abnormalities in the brain that are implied in various pathological processes. Among various imaging modalities, magnetic resonance imaging (MRI) is advantageous in examining the brain anatomy compared to computerized tomography (CT) which provides limited signal contrasts for soft tissue. MRI, by utilizing a blood-oxygenation-level-dependent (BOLD) endogenous contrast mechanism associated with local brain activations, can also reveal spatiotemporal pattern of regional brain function in response to external stimulation in anesthetized animals (referred as functional MRI; fMRI).

MRI also offers information on the white matter (WM) structures and their spatial orientations. Diffusion tensor MRI (DTI), introduced in the mid 90s [8, 9], uses multiple diffusion-sensitive pulsed-gradient pairs with differing directions that provide characterization of the diffusion of proton-bearing molecules along three orthogonal directions in each voxel. Computed diffusion tensor eigenvectors represent the voxel-specific orthogonal principal axes of diffusion, in which each respective eigenvalue reflects the amount of diffusivity in these axes [10]. Apparent diffusion can then be quantitatively analyzed, conveying intra-voxel information about the averaged local (intracellular and extracellular) water diffusion in the brain tissue. Combined with tractography technique [11–13], therefore, DTI offers information on macroscopic orientations in the WM tracts.

While these techniques have well been established and widely used in humans [14, 15] and animals (such as rats [16, 17], cats [18, 19], or dogs [20, 21], the application in ovine has rarely been performed. Opdam *et al.* [22] showed concurrent fMRI and EEG data acquisition in a sheep epilepsy model to localize the epileptic locus. Boltze *et al.* [3] used MRI and PET along with CT for neuroimaging of a sheep stroke model. A recent work by Hoffmann *et al.* [23] presented a combination of anatomical MRI and CT data along with cranial anatomy, focusing on the ovine cerebrovascular system. While these previous studies showed the potential utilization of neuroradiological imaging in ovine, only a limited amount of information on the healthy brain was available. Therefore, we were motivated to examine the feasibility of applying a routine human neuro MRI protocol to image the normal sheep brain. Anatomical MRI as well as functional MRI (fMRI) was conducted during photic stimulation (presented through closed eyelid) and tactile stimulation (on the unilateral hind leg). The photic and tactile stimulations are some of the earliest stimulation modalities used in the context of fMRI, and are widely adopted in studies involving humans [24–27] and small animals [28, 29]. Because these stimulation modalities can activate the primary sensory circuities (such as primary sensory and visual areas) even during the anesthesia [28, 30], they are compatible with animal studies. Besides fMRI, voxel-wise apparent diffusion coefficient (ADC), along with fractional anisotropy (FA; as a measure of the directionality of diffusion) values were obtained using DTI, and the spatial orientations of the corticospinal tracts and optic radiations were visualized using tractography technique.

## Results

### Example of individual T1, T2, fMRI, and 3D SPGR high resolution MRI rendering

**Fig. 1:**
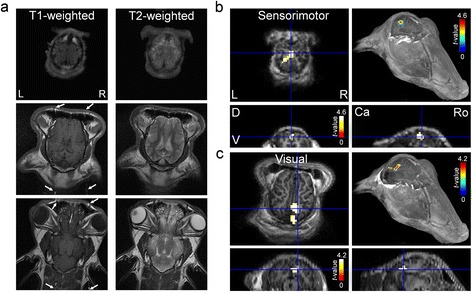
Example of MRI data from an individual sheep. **a** T1-weighted (left column) and T2-weighted (right column) axial images from dorsal (top) to ventral (bottom). In the T1 images, arrows indicate flow-related artifacts along the phase-encoding direction. Individual fMRI activation maps from (**b**) the sensorimotor and (**c**) visual stimulations were overlaid on axial, coronal, and sagittal views as well as on a 3D MRI head rendering. Crosshairs (in blue) indicate the location of the local maxima on the fMRI activation map from the square frame (in blue) showed on an axial plane of the 3D rendering. L: left, R: right, D: dorsal, V: ventral, Ca: caudal, and Ro: rostral

All animals successfully underwent the MRI session. Exemplar T1- and T2-weighted axial images of the sheep brain are shown in Fig. 1a. In the T1 images, small vascular flow artifacts were visible along the phase-encoding direction (marked with arrows) due to the vascular anatomy on ovine facial area (angularis oculi vein [31, 32]). The activated functional areas, from the sensorimotor stimulation (Fig. 1b) and visual stimulation (Fig. 1c) of one sheep, were visualized (uncorrected *P* < 0.001, with a cluster extent threshold of 27 continuous voxels) and overlaid on axial, coronal, and sagittal slices, along with a 3D-rendered head view.

### fMRI group processing results and the time-course of BOLD signals

**Fig. 2:**
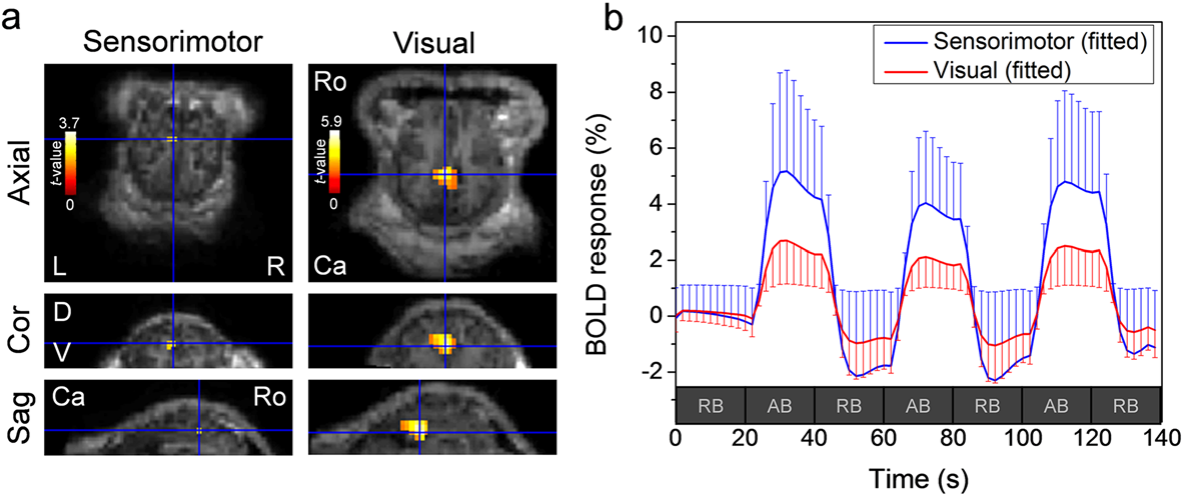
Group-processed fMRI activation maps and the time-course of BOLD signals. **a** Group-level fMRI activation maps from seven sheep *via* random-effect analysis of the sensorimotor area (*P* < 0.05, no voxel-based corrections) and visual area (uncorrected *P* < 0.01, with cluster extent threshold of 27 contiguous voxels) were overlaid on axial, coronal and sagittal anatomical MRI. Left column: *via* sensorimotor stimulation, Right column: *via* visual stimulation. L: left, R: right, D: dorsal, V: ventral, Ca: caudal, and Ro: rostral. **b** The time-course of BOLD responses averaged across the group of sheep (*n* = 7, error bars are standard deviation of the signal) from the local maxima of sensorimotor-(blue) and visual-(red) related region-of-interests (ROIs) on fMRI activation maps. RB: resting block (20 s-long), AB: activation block (20 s-long)

fMRI group processing results (*n* = 7) that identified the activated sensorimotor (*P* < 0.05, no voxel-based corrections) and visual areas (uncorrected *P* < 0.01, with a cluster extent threshold of 27 continuous voxels) are shown in Fig. 2a. The location and the size of the activated regions from the group analysis according to the different statistical properties (with and without the extent threshold correction) were tabulated (Table 1). Due to the small SM1 area for the hind leg among sheep [33], no additional extent correction was performed when thresholded at *P* < 0.05. The activation map was overlaid and visualized on axial, coronal, and sagittal slices of the high resolution 3D SPGR image of an individual sheep. The time-courses of BOLD signals (averaged across seven sheep) from the sensorimotor and visual ROIs are shown in Fig. 2b. The variabilities in spatial distribution of activation among sheep (*n* = 7), as measured from the coordinates showing the local maximum in the voxel-wise *t*-value, were quantified by taking the standard deviation along the left-right, rostral-caudal, and dorsal-ventral directions (5.40, 6.25, and 5.70 mm for the sensorimotor area, and 5.32, 3.93, and 3.40 mm for the visual area). The time delay of the observed BOLD signal was approximated at 3–4 s from the onset of the stimulation with the magnitude of ~3 % (in case of the sensorimotor stimulation) and ~2 % (in case of the visual stimulation) of the baseline signal level.

**Table 1:** The location and the size of the activated regions from group analysis (*n* = 7) with the different statistical properties

	Sensorimotor stimulation		Visual stimulation	
P-value	Activated region	Voxel size	Activated region	Voxel size
0.05 (Uncorrected)	SM1	3	V1	212
0.05 (Corrected)	-	0	V1	212
0.01 (Corrected)	-	0	V1	36

Corrected: application of a cluster extent threshold of 27 continuous voxels, SM1: primary sensorimotor area, V1: primary visual area

### FA and ADC values and tractography of corticospinal tracts in sheep

**Fig. 3:**
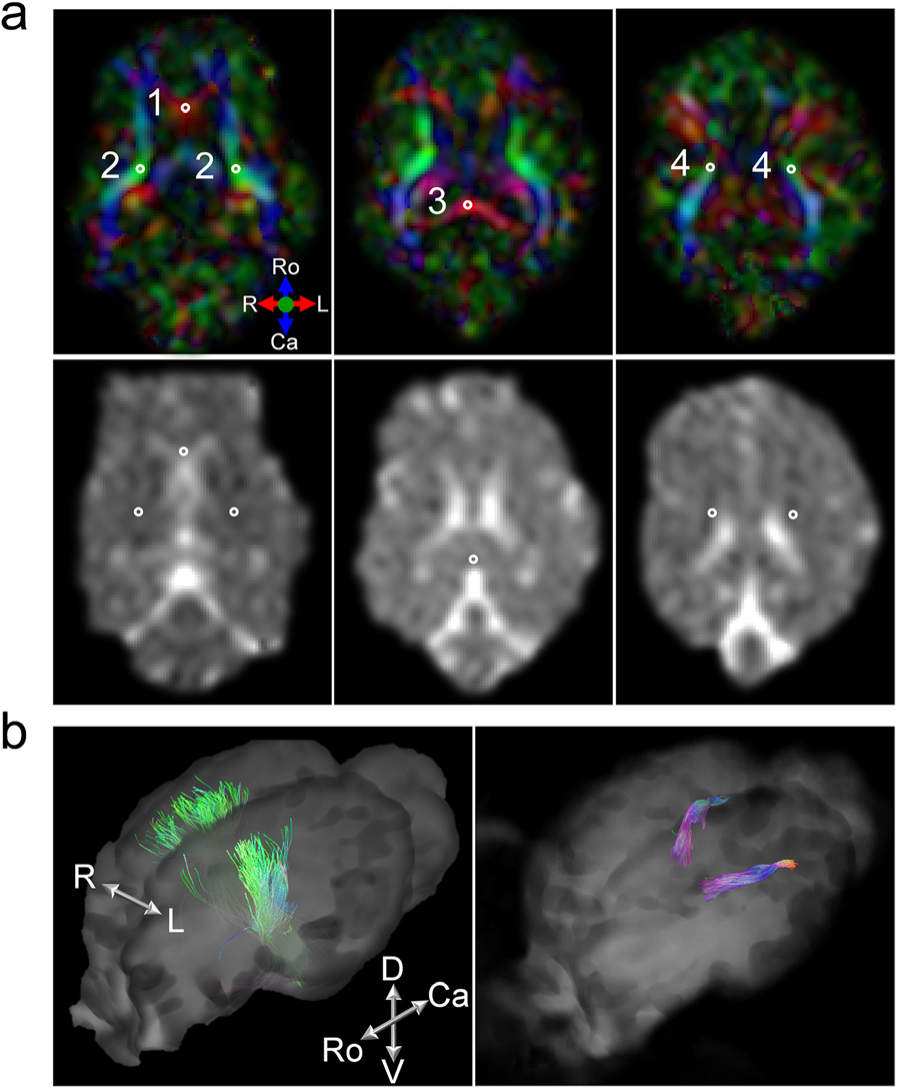
The processed DTI data to visualize white matter tracts in the brain. **a** The placement of the regions of interests (ROIs) on the color-coded fractional anisotropy (FA) map and the corresponding apparent diffusion coefficient (ADC) map. #1: genu of corpus callosum, #2: internal capsule, #3: splenium of corpus callosum, #4: corona radiate. The regions outside of the brain parenchyma were masked out from the display. For the FA color-coding scheme, red, green, and blue colors were used for left–right, dorsal–ventral, and rostral–caudal directions, respectively. **b** Bilaterally visualized DTI tractography of the corticospinal tracts and optic radiations in sheep (software-generated pseudo-coloring was used)

The calculated color-coded FA maps, showing the fiber orientations (Fig. 3a, upper low), and ADC maps (Fig. 3a, lower low) were shown in axial view, along with the selected ROIs used to measure the values. For the color-coding scheme, red, green, and blue colors were used for left–right, dorsal–ventral, and rostral–caudal directions, respectively. The mean FA and ADC values measured from the selected ROIs were tabulated in Table 2.

**Table 2:** Regional mean values of fractional anisotropy (FA) and apparent diffusion coefficient (ADC)

(a) Sheep 3 T (*n* = 6)				(b) Human 3 T (*n* = 30)			
		FA	ADC			FA	ADC
		×10^−3^ mm^2^/s				×10^−3^ mm^2^/s	
Region		Mean ± SD	Mean ± SD	Region		Mean ± SD	Mean ± SD
CC genu		0.39 ± 0.05	1.14 ± 0.16	CC genu		0.84 ± 0.03	0.76 ± 0.04
CC splenium		0.37 ± 0.16	0.91 ± 0.07	CC splenium		0.86 ± 0.05	0.71 ± 0.03
Internal capsule	Left	0.63 ± 0.13	0.84 ± 0.21	Internal capsule	Left	0.75 ± 0.04	0.68 ± 0.03
	Right	0.65 ± 0.16	0.75 ± 0.12		Right	0.75 ± 0.05	0.70 ± 0.03
Corona radiata	Left	0.48 ± 0.13	0.80 ± 0.07	Corona radiata	Left	0.52 ± 0.05	0.65 ± 0.04
	Right	0.46 ± 0.18	0.89 ± 0.14		Right	0.48 ± 0.04	0.66 ± 0.03

FA and ADC from (a) sheep DTI data (*n* = 6) and (b) human DTI data (adapted from Brander *et al*. [46]), acquired with 3 T MRI. CC: corpus callosum

In the corpus callosum (CC) genu (Fig. 3a, ROI #1), the FA value was 0.39 ± 0.05 (mean ± SD) and the corresponding ADC value was 1.14 ± 0.16 × 10^−3^ mm^2^/s (mean ± SD). The FA value measured from the CC splenium (Fig. 3a, ROI #3) was 0.37 ± 0.16 and the ADC value was 0.91 ± 0.07 × 10^−3^ mm^2^/s. The greatest FA values were observed from the internal capsules (Fig. 3a, ROI #2), which were 0.63 ± 0.13 for the left and 0.65 ± 0.16 for the right. The corresponding mean ADC values were 0.84 ± 0.21 × 10^−3^ and 0.75 ± 0.12 × 10^−3^ mm^2^/s. From the ROIs for the corona radiata (Fig. 3a, ROI #4), the mean FA values were 0.48 ± 0.13 for the left and 0.46 ± 0.18 for the right, while the corresponding mean ADC values were 0.80 ± 0.07 × 10^−3^ and 0.89 ± 0.14 × 10^−3^ mm^2^/s, respectively. In the analysis of interhemispheric differences, no significant differences were found in both FA and ADC values in the internal capsule (paired *t*-test, two-tailed, *P* = 0.67 for FA, *P* = 0.20 for ADC, *n* = 6) or from the corona radiata (paired *t*-test, two-tailed, *P* = 0.60 for FA, *P* = 0.21 for ADC, *n* = 6). DTI tractography performed on the DTI data visualized the corticospinal tracts and optic radiations bilaterally (Fig. 3b), with the seeding areas of the internal capsule and thalamic lateral geniculate areas for the tracking, respectively.

## Discussion

The present study demonstrated the applicability of MR imaging for non-invasive neuro-functional assessment in a healthy sheep model. fMRI was employed to examine the sensorimotor and visual cortex activated by external sensory stimuli in healthy sheep. The identified functional areas were in a good agreement with the areas probed by using cortical stimulation of the motor area [33] and visual photic stimulation [34]. Also, the measurements taken from DTI (FA and ADC values) as well as the tractography showed the feasibility of examining the WM macrostructures.

The time-course of BOLD signals obtained from the sheep (Fig. 2b) showed contrast magnitude on the order of 2–3 % (from the resting signal level) and an onset response delay of 3–4 s, exhibiting similarities to the signals from humans [35–37]. Since the current fMRI protocol (with TR of 2 s) lacks the temporal precision to produce an accurate representation of the signal response delay, a study employing the use of a shorter TR (on the order of less than 1 s) combined with the event-related designs [38, 39] would reveal more comprehensive information on the BOLD hemodynamic properties from the sheep. It is also important to note that the BOLD response is affected by the anesthetic depth or by type of anesthetic agents due to potential alterations of the hemodynamic response to the neuronal activation [40–42]. Further study is warranted to examine the effects of different anesthetic agents, other than Telazol, such as propofol infusion [43–45], on the BOLD signal responses and the central nervous system in ovine species.

In the interpretation of the DTI data, both FA and ADC values from the ROIs in sheep (the CC genu and splenium, and the internal capsule, shown in Fig. 3a) were qualitatively comparable to the data from healthy human adults reported by Brander *et al.* [46] (Table 2). On the other hand, the FA values measured from sheep’s corona radiata (left: 0.48 and right: 0.46) were comparable to those obtained from humans (left: 0.52 and right: 0.48) [46]. In the interhemispheric differences in sheep, there were no significant differences in both FA and ADC values from the internal capsule and the corona radiata, while significant left/right asymmetries from those regions have been reported in humans [46].

Additionally, the FA values from the CC genu and splenium, which are typically shown to be greater than the values from the internal capsule and the corona radiata among humans [46], were smaller than the values from other ROIs (Fig. 3a). We also noted greater ADC values from these areas compared to the ones from humans. We conjecture that these discrepancies may be attributed to the partial volume effects caused by the relatively thick imaging slice (3 mm) and large voxel volume with respect to the ovine neuroanatomy.

DTI tractography successfully visualized the corticospinal tracts and optic radiations in sheep (Fig. 3b), but was inadequate to visualize other WM tracts such as the frontoparietal fasciculus, which are readily characterized through the human version of the imaging protocol. The insufficient image-resolution (slice thickness on the order of 3 mm) and signal-to-noise ratio (SNR) were considered as the causes of the limited ability to track major WM tracts. The use of a head coil that is designed for a human head might also have resulted in the lack of SNR of the acquired neuroanatomical data (due to smaller voxel size compared to the human counterparts due to use of smaller field-of-view). The use of higher spatial resolution (*i.e.,* smaller voxel size, ideally isotropic in volume) combined with a dedicated head coil having smaller coil elements to boost SNR (to compensate for the decrease in the MR signal associated with the higher spatial resolution) would be conducive to identifying small WM bundles in sheep. The use of a sheep-dedicated head coil would not only improve the SNR but would also provide more homogenous signal sensitivity profile across the brain volume.

In the review of overall experimental procedures, we experienced a few technical difficulties, especially from the head movement of the anesthetized sheep during the MRI session(s), which considerably reduced the quality of the image in couple of animals. Although restrainers placed over the sheep head (*i.e.,* use of cushion between the sheep head and a head coil) were helpful in reducing the artifacts, bloating of the abdomen could have amplified breathing-related head motion beyond the restraining capacity. Since ruminants are especially susceptible to bloating, the use of intubation would be beneficial to prevent the bloating of stomach during imaging procedures.

We also noted the artifacts in T1 images from the sheep (Fig. 1a, arrows), which were likely attributable to the vascular feature (angularis oculi veins [31, 32]) that prominently exists in the ovine facial area but not in humans. The artifacts call for necessary countermeasures, such as introduction of additional flow-suppression [47] or cardiac gating [48], when using human-compatible imaging protocol on ovine species.

## Conclusions

Ovine model can provide a unique opportunity for testing new neurotherapeutic modalities that can potentially be translated to clinical applications in humans, for example, to focused ultrasound (FUS) brain stimulation technology which has shown evidences in neuromodulatory potentials in small animal models [1]. Applicability of MRI, fMRI, and DTI in the sheep brain, as demonstrated in the present study, could further enhance the range of utilization of an ovine model in the field of translational neuroscientific research targeting the brain.

## Methods

### Overview

The study was conducted under the approval of the Harvard Medical Area Standing Committee on Animals. Sheep (Dorset, all female, 20–40 kg, *n* = 8) were anesthetized with Telazol (Tiletamine; N-methyl-D-aspartate; NMDA receptor antagonist + zolazepam, initial dose 2–4 mg/kg *i.v.* + additional doses as needed) for the MRI procedures. The use of inhalant anesthetics, such as isoflurane, was not chosen due to its effects on altering the cerebral blood hemodynamic responses, such as vasodilation [49, 50], which confound the BOLD signal. The adequacy of anesthesia was assessed by the absence of the reflex withdrawal of the hind limb in response to pinching, and by monitoring the heart rate and peripheral capillary oxygen saturation level (SpO_2_) during the procedure. The anesthetic agent was administered only to those animals with normal vital signs. Cushions were placed between the sheep head and a head coil to discourage movement and subsequent motion-related MR artifacts.

### Data acquisition

MRI was performed to obtain the anatomical information of the sheep brain. A 3 tesla clinical MRI scanner (GE VH, Waukesha, WI) with an 8-channel head coil was used. Anatomical T1- and T2-weighted images (spin echo; SE for T1 and fast spin echo; FSE for T2, field-of-view; FOV = 18 × 18 cm^2^, slice thickness = 3 mm, image matrix = 512 × 512, voxel size = 0.35 × 0.35 × 3 mm^3^, TR/TE = 567/9 ms for T1 and TR/TE = 2783/100 ms for T2, flip angle = 65 °) were acquired as sections of the ventral–dorsal orientation with respect to the animal’s head covering the entire brain area. To provide high resolution anatomical information, we also acquired volumetric T1-weighted images covering most of the head including the brain (inversion recovery; IR – 3D spoiled gradient recalled sequence; SPGR, orientation in 3D blocked sagittal, FOV = 22 × 22 cm^2^, slice thickness = 1 mm, image matrix = 256 × 256, voxel size = 0.86 × 0.86 × 1 mm^3^, TR/TE = 7/3 ms, flip angle = 11 °).

Functional MRI (fMRI) was performed to map the sensorimotor and visual areas of the sheep brain, using gradient-echo echo-planar-imaging (EPI) sequence (TR/TE = 2000/40 ms; flip angle = 90°; FOV = 18 × 18 cm^2^; image matrix = 64 × 64; slice thickness = 3 mm; slices = 20, no gap; voxel size = 2.81 × 2.81 × 3.00 mm^3^) to image the brain as sections of the ventral–dorsal orientation with respect to the animal’s head for obtaining the T2*-weighted blood-oxygenation-level-dependent (BOLD) signal. Although TE of 30–35 ms is widely used in fMRI at 3 T environment, a slightly longer TE was used in this study to maximize the sensitivity toward the BOLD contrast from the sheep [51]. The scanning orientation was set using the same scan prescription location as the anatomical T1- and T2-weighted brain images. The activations of the sensorimotor and visual areas were evoked using a passive tactile stimulation (*i.e.,* gentle 2 Hz squeeze of the right hind leg muscle) and a photic stimulation (*i.e.,* 2 Hz white strobe lights to both eyes with eyelids closed), respectively. Three blocks of the stimulation period (20 s, synchronized with the scanner operation) were interleaved by four resting periods of equal duration (20 s). A dummy scan of 20 s was included in the beginning of the imaging session to allow for T1 signal equilibration, but was not used in the data processing.

A diffusion tensor imaging (DTI) sequence (spin echo; SE, echo-planar-imaging; EPI, oblique axial orientation, FOV = 18 × 18 cm^2^, slice thickness = 3 mm, image matrix = 256 × 256, voxel size = 0.7 × 0.7 × 3.0 mm^3^, TR/TE = 6000/90 ms, flip angle = 90°, acceleration factor = 2; 26 gradient directions, b-factor = 1000) was used to examine FA and ADC values of the sheep brain. 26 non-overlapping gradient directions were used to probe the spatial orientation of voxel-wise water diffusivity. All imaging parameters were similar to the ones used in an adult human, however, a smaller FOV (*i.e.,* 18 × 18 cm^2^) was used compared to the one used in human studies (*i.e.,* 22 × 22 cm^2^).

### fMRI data processing

The fMRI time series from seven sheep were used for the data processing. Data from one sheep were excluded due to severe motion-related artifacts (> 5 mm in translational motion) during the scan. The time series were processed by the SPM8 software package (Wellcome Department of Imaging Neuroscience, University College London, London, UK; www.fil.ion.ucl.ac.uk/spm). The datasets obtained from the group of sheep underwent slice time correction, and were neuroanatomically co-aligned for motion correction. Then, the datasets were spatially smoothed with the Gaussian kernel having a full-width at half maximum (FWHM) of [8 × 8 × 8] in mm. After the motion-correction and smoothing, the task-related neuronal activity was estimated by a general linear model (GLM) for each individual sheep. The degree of voxel-wise statistic parametric map in *t*-value, with respect to the task-specific canonical hemodynamic response function (HRF), was obtained. To derive the group-averaged trend, random effect analysis (RFX) [52] was used to perform a statistical test at the group level. To reject false positives based on cluster extent thresholds [53] in the statistical parametric map, the cluster size was calculated using the REST AlphaSim toolbox [54] (www.restfmri.net; toolkit v1.8; 1000 Monte Carlo simulations were used). The time-course of BOLD signals from the region-of-interests (ROIs) for the sensorimotor and visual areas were averaged across the animals (*n* = 7).

### DTI data processing

Data from two animals, one due to motion effects and the other due to technical issues, were excluded. The acquired DTI sequence (*n* = 6) was processed by the DTI Studio software (Department of Radiology, Johns Hopkins University, Baltimore, MD), whereby diffusion tensor calculation was performed with diffusion-weighted image parameters (slice orientation: axial, slice sequencing: inferior-superior, b-value = 1000 s/mm^2^). Fractional anisotropy (FA), apparent diffusion coefficient (ADC), trace, tensor, and color-coded directional FA maps were calculated as provided in the software. FA and ADC are one of the most frequently used quantitative parameters in humans. Circular region-of-interests (ROIs), having a size of 9 pixels, were delineated based on the directional FA maps. The ROIs were placed on the following anatomical locations (also shown in Fig. 3a): on the genu and splenium of the corpus callosum (CC), on the left and right internal capsule, and on the left and right corona radiate, and the FA and ADC values from these ROIs were averaged (Table 2). The ADC value was calculated as the trace of the tensor (Tr) divided by three (ADC = Tr/3) [55]. To demonstrate the feasibility of DTI tractography, white matter tracking routine, as implemented by the MRtrix [56, 57], was employed using the software options (start tracking criteria FA > 0.3; stop tracking criteria FA < 0.3 and fiber turning angular tolerance of 20 °). The seeding areas were defined in the internal capsule and thalamic lateral geniculate areas for the tracking and visualization of the corticospinal tracts and optic radiations, respectively.

## Abbreviations

ADC: Apparent diffusion coefficient
BOLD: Blood-oxygenation-level-dependent
CC: Corpus callosum
CT: Computerized tomography
DTI: Diffusion tensor MRI
EPI: Echo-planar-imaging
FA: Fractional anisotropy
fMRI: Functional MRI
FWHM: Full-width at half maximum
GLM: General linear model
HRF: Hemodynamic response function
MRI: Magnetic resonance imaging
RFX: Random effect analysis
ROI: Region-of-interest
SNR: Signal-to-noise ratio
TE: Echo time
TR: Repetition time
WM: White matter
